# Efficacy of Jishi Shuanghua Granules in preventing radiation-induced esophagitis in non-small cell lung cancer patients undergoing concurrent chemoradiotherapy: protocol for a multicenter, randomized, double-blind, placebo-controlled trial

**DOI:** 10.3389/fmed.2026.1814342

**Published:** 2026-06-23

**Authors:** Huakang Li, Yuanzhen Mi, Ke Xu, Ming Fan, Jun Yin, Qiang Li, Ziliang Wu, Cuicui Gong, Yunjing Jia, Pengxuan Gu, Shanshan Wei, Zhonglin Zhang, Shuo Zhang, Yuanyuan Zheng, Bing Lin, Jinyi Lang, Biao Zhao, Meihua Chen

**Affiliations:** 1 School of Clinical Medicine, Chengdu University of Traditional Chinese Medicine, Chengdu, Sichuan, China; 2 Department of Radiation Oncology, Precision Radiation in Oncology Key Laboratory of Sichuan Province, Sichuan Clinical Research Center for Cancer, Sichuan Cancer Hospital and Institute, Sichuan Cancer Center, University of Electronic Science and Technology of China, Chengdu, China; 3 Department of Medical Oncology, Affiliated Hospital of Sichuan Nursing Vocational College, The Third People’s Hospital of Sichuan, Chengdu, Sichuan, China; 4 Department of Medical Oncology, Hospital of Chengdu University of Traditional Chinese Medicine, Chengdu, China; 5 Division of Internal Medicine, Department of Integrated Traditional Chinese and Western Medicine, Leshan City Shizhong District Cancer Hospital, Leshan, Sichuan, China; 6 Health Management Center, Hospital of Chengdu University of Traditional Chinese Medicine, Chengdu, China; 7 Department of Integrative Chinese and Western Medicine, Sichuan Cancer Center, Sichuan Cancer Hospital and Institute, Affiliated Cancer Hospital of the University of Electronic Science and Technology of China, Chengdu, China

**Keywords:** concurrent chemoradiotherapy, non-small cell lung cancer, radiation-induced esophagitis, Traditional Chinese Medicine, trial protocol

## Abstract

**Background:**

Acute radiation-induced esophagitis (ARIE) is a common complication in non-small cell lung cancer (NSCLC) patients undergoing concurrent chemoradiotherapy (CCRT), negatively impacting their quality of life and treatment outcomes. Jishi Shuanghua Granules (JSG), a Traditional Chinese Medicine formulation, is widely used in clinical practice and has received positive feedback. However, high-quality evidence is still lacking. This study aims to evaluate the efficacy of JSG in reducing ARIE risk and severity and to explore its mechanisms through a multicenter, randomized, double-blind, placebo-controlled trial.

**Methods:**

A total of 240 stage III NSCLC patients scheduled to undergo definitive CCRT will be recruited and randomly assigned in a 1:1 ratio to either the JSG or placebo group. The primary efficacy endpoints will be the incidence rates of ARIE (≥Grade 1) and severe ARIE (SARIE, ≥Grade 3) based on the Radiation Therapy Oncology Group (RTOG) criteria. Secondary efficacy endpoints will include time to first occurrence of ARIE and SARIE, ARIE-related pain and dysphagia (assessed using the Numerical Rating Scale), quality of life (measured by the Functional Assessment of Cancer Therapy-Lung questionnaire), short-term lung cancer efficacy (objective response rate and disease control rate), and long-term outcomes (progression-free survival and overall survival). Additionally, the study will explore the mechanisms of JSG through multi-omics analyses, including lymphocyte subsets, inflammatory cytokines, oxidative stress markers, gut microbiota, and metabolomics.

**Discussion:**

If the trial meets its expected outcomes, this study will provide high-quality evidence supporting the use of JSG in preventing and treating ARIE. Furthermore, integrating multi-omics analyses will help elucidate the multi-target mechanisms of JSG, contributing to a more comprehensive evidence base.

**Clinical trial registration:**

http://itmctr.ccebtcm.org.cn/, identifier [ITMCTR2025000641].

## 1 Introduction

Lung cancer remains the leading cause of cancer-related mortality globally, with non-small cell lung cancer (NSCLC) constituting approximately 80%–85% of all lung cancer cases (1). For patients with unresectable stage III NSCLC, concurrent chemoradiotherapy (CCRT) represents the current standard of care (2). Compared with radiotherapy alone or sequential chemoradiotherapy, CCRT significantly enhances local control rates and overall survival but concurrently elevates the risk and severity of acute radiation-induced esophagitis (ARIE) (3). Approximately 95% of NSCLC patients undergoing CCRT experience ARIE to varying degrees, with severe ARIE (SARIE, grade ≥ 3) occurring in 18%–32% of cases (3, 4). SARIE is characterized by persistent severe pain and dysphagia, frequently necessitating nutritional interventions, such as nasogastric tube insertion or total parenteral nutrition. This not only significantly diminishes patient quality of life (QoL) but may also compel clinicians to modify CCRT regimens, including treatment interruption or dose reduction, thereby adversely affecting patient prognosis (5).

Although ARIE has garnered attention as a significant complication of CCRT, there remains a clear unmet medical need in its prevention and treatment. In terms of prevention, although various potential radioprotective agents such as amifostine and glutamine have been extensively investigated, there is still a lack of drugs supported by definitive evidence of efficacy and safety (6). In terms of treatment, current therapeutic approaches continue to focus primarily on symptomatic management. In Chinese clinical practice, ARIE is often managed with compound oral solutions containing local anesthetics, glucocorticoids, B vitamins, and antibiotics to alleviate pain and inflammation, one of the more commonly used formulations being a mixture of lidocaine, dexamethasone, and vitamin B12 (mLDV).

With the advancement of integrative medicine, Traditional Chinese Medicine (TCM), characterized by its multi-target and multi-pathway mechanisms, has garnered increased attention in ARIE management. According to TCM theory, ARIE is predominantly caused by excessive heat, and “clearing heat” is the foundational therapeutic principle. A systematic review comprising 11 randomized controlled trials indicated that herbal formulations guided by the “clearing heat” principle could reduce the risks of ARIE and SARIE by approximately 16% and 59%, respectively (7). However, the review also noted that all included studies were published in Chinese and were generally limited by small sample sizes, low methodological quality, high risk of bias, and inconsistent reporting, underscoring the need for high-quality research to validate these findings.

Jishi Shuanghua Granules (JSG) is a TCM herbal formulation developed by our team based on traditional theories, literature review, and clinical experience, and has been clinically utilized for managing CCRT-induced ARIE for over a decade. This formulation comprises 12 herbal ingredients (Table 1), predominantly featuring nine heat-clearing herbs (Gypsum Fibrosum, Lonicerae Japonicae Flos, Trichosanthis Radix, Arnebiae Radix, Glycyrrhizae Radix et Rhizoma, Rehmanniae Radix, Scrophulariae Radix, Ophiopogonis Radix, and Glehniae Radix) complemented by three hemostatic herbs (Bletillae Rhizoma, Notoginseng Radix, and Typhae Pollen). All components are included in the Chinese Pharmacopeia (2020 edition), complying with rigorous standards for legality, safety, and efficacy. Previous studies have demonstrated that the individual herbal components of JSG exert multiple pharmacological effects (8–24) (Table 1). These activities may act synergistically to target several critical pathological processes underlying ARIE development, including immunosuppression, inflammation, oxidative stress, and disruption of mucosal integrity.

**TABLE 1:** Standard formulation and mechanism of Jishi Shuanghua Granules (JSG).

Chinese name	English name	Latin name	Granule dose (crude drug equivalent)	Pharmacological activities
Shigao	Gypsum	*Gypsum Fibrosum*	0.48 g (6 g)	Anti-inflammatory, antioxidant, antiviral, and immunity-enhancing activities (8)
Jinyinhua	Wild honeysuckle flower	*Lonicerae Japonicae Flos*	2.00 g (6 g)	Anti-inflammatory, antioxidant, anticancer, antibacterial, and antiviral activities (9)
Tianhuafen	Mongolian Snakegourd Root	*Trichosanthis Radix*	1.11 g (5 g)	Anti-inflammatory and antioxidant activities (10)
Zicao	Gromwell root	*Arnebiae Radix*	0.30 g (3 g)	Anti-inflammatory, antiviral, and anticancer activities (11, 12)
Sheng Gancao	Liquorice root	*Glycyrrhizae Radix et Rhizoma*	1.00 g (3 g)	Anti-inflammatory, anticancer, antibacterial, and antioxidant activities (13)
Sheng Dihuang	Rehmannia glutinosa	*Rehmanniae Radix*	2.86 g (4 g)	Anti-inflammatory, antioxidant, anticancer, immunomodulatory, and immunoenhancing effects (14, 15)
Xuanshen	Figwort root	*Scrophulariae Radix*	2.67 g (4 g)	Anti-inflammatory, immunomodulatory, and antioxidant activities (16)
Maidong	Dwarf lilyturf tuber	*Ophiopogonis Radix*	3.64 g (4 g)	Anti-inflammatory, antioxidant, anticancer, immunomodulatory, and antimicrobial activities (17)
Beishashen	Coastal Glehnia Root	*Glehniae Radix*	1.60 g (4 g)	Anti-inflammatory, antiviral, anticancer, and immunomodulatory activities (18, 19)
Baiji	Common bletilla tuber	*Bletillae Rhizoma*	1.00 g (5 g)	Promotes wound healing, hemostatic, anti-inflammatory, antioxidant, and immune-regulating activities (20)
Puhuang	Cattail pollen	*Typhae Pollen*	0.60 g (3 g)	Hemostatic, antioxidant, and immunomodulatory activities (21, 22)
Sanqi	Sanchi	*Notoginseng Radix*	1.33 g (2 g)	Hemostatic, anticancer, promotes wound healing, anti-inflammatory, antioxidant, antimicrobial, and improvement of microcirculatory disorders (23, 24)

Although JSG has been widely used in clinical practice and has shown positive feedback, rigorous evidence from well-designed studies remains scarce. To fill this research gap, we have designed this multicenter, prospective, randomized, double-blind, placebo-controlled clinical trial protocol to systematically evaluate the efficacy and safety of JSG in reducing the risk and severity of ARIE. Given the multi-target and multi-pathway nature of TCM, we will also integrate multiomics analyses, including lymphocyte subsets, inflammatory cytokines, oxidative stress markers, gut microbiota, and metabolomics, to elucidate the underlying mechanisms of JSG and construct a comprehensive evidence chain. We anticipate that our findings will further support the modern translational application and wider clinical adoption of the “clearing heat” strategy for ARIE management.

## 2 Methods

### 2.1 Study design

**FIGURE 1:**
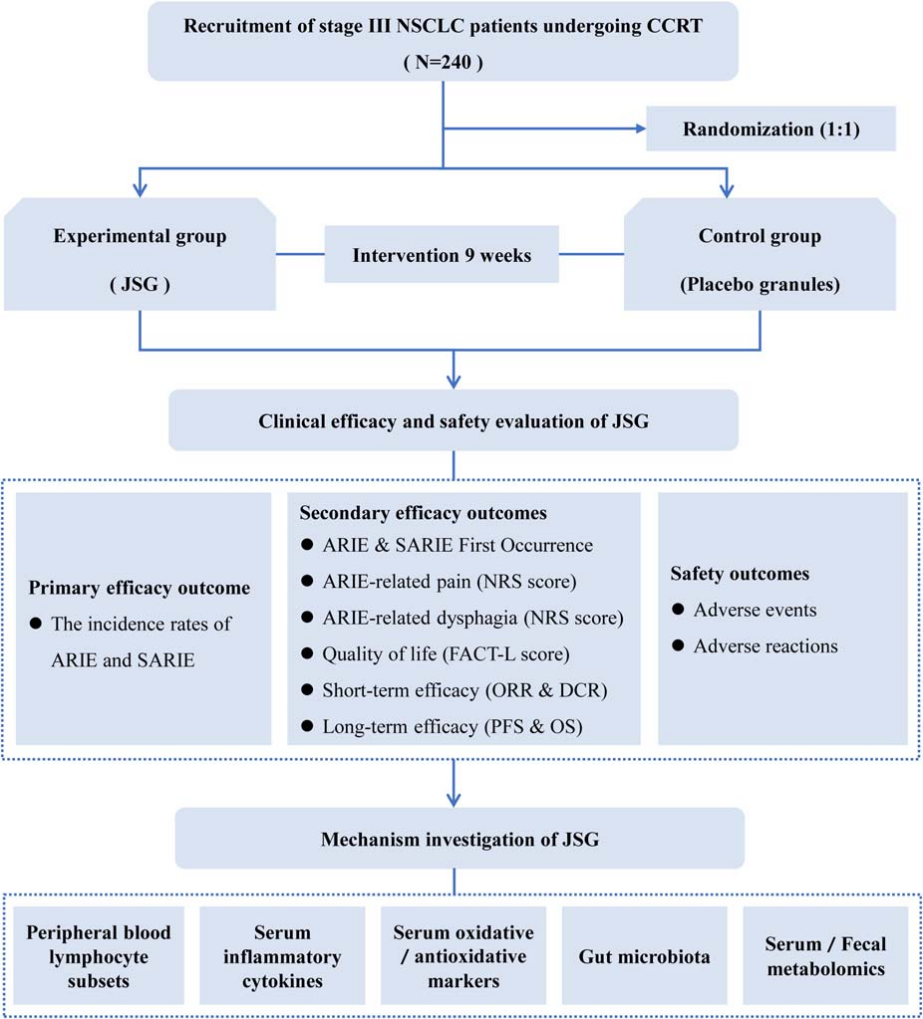
Study flowchart. ARIE, acute radiation-induced esophagitis; CCRT, concurrent chemoradiotherapy; DCR, disease control rate; FACT-L, Functional Assessment of Cancer Therapy-Lung; JSG, Jishi Shuanghua granules; NSCLC, non-small cell lung cancer; NRS, numerical rating scale; ORR, objective response rate; OS, overall survival; PFS, progression-free survival; SARIE, severe acute radiation-induced esophagitis.

The study is designed as a multicenter, prospective, randomized, double-blind, placebo-controlled clinical trial with two parallel groups. A total of 240 patients with stage III NSCLC undergoing CCRT will be recruited from four participating centers: Sichuan Cancer Hospital, Hospital of Chengdu University of TCM, West China Hospital of Sichuan University, and Leshan City Shizhong District Cancer Hospital. Participants will be randomized in a 1:1 ratio to either the experimental group (receiving JSG) or the control group (receiving placebo granules). The primary efficacy outcomes are the incidence rates of ARIE and SARIE from the initiation of CCRT to 4 weeks after completion of CCRT, corresponding to baseline to week 11. This study protocol has been meticulously designed in accordance with the CONSORT guidelines and is reported following the 2013 SPIRIT Checklist (25) (Supplementary File 1). The study flowchart is presented in Figure 1. The study protocol was approved by the Ethics Committee of Leshan City Shizhong District Cancer Hospital (approval number: EC-2025-001). The ethical approval has been recognized by the other three participating centers. The trial was registered at the International Traditional Medicine Clinical Trial Registration Center (ITMCTR2025000641) on April 2, 2025.

### 2.2 Inclusion criteria

The inclusion criteria are as follows: (1) age between 18 and 75 years; (2) pathologically confirmed NSCLC, classified as stage III (according to the 8th edition of the American Joint Committee on Cancer staging system); (3) clinically assessed as unresectable and scheduled to undergo definitive CCRT; (4) mean esophageal radiation dose > 20 Gy; (5) normal hematologic, hepatic, and renal function (based on laboratory reference ranges); and (6) Karnofsky Performance Status (KPS) score ≥ 70.

### 2.3 Exclusion criteria

The exclusion criteria are as follows: (1) prior history of thoracic radiotherapy; (2) pregnancy or lactation; (3) inability to maintain adequate oral intake; and (4) history of esophageal diseases, including but not limited to esophageal cancer, active reflux esophagitis, or esophageal reconstruction.

### 2.4 CCRT

All participants will receive definitive CCRT. Radiation therapy will be administered using intensity-modulated radiation therapy, with a total radiation dose of 60–70 Gy (delivered at 2.0–2.4 Gy per fraction, once daily, 5 days per week) over a treatment period of 6–7 weeks. The radiotherapy protocol will be consistent with the methods detailed in previous studies (26). The concurrent chemotherapy regimen will be a platinum-based doublet regimen, with platinum options including cisplatin (25 mg/m^2^ on days 1–3) or carboplatin (area under the curve = 5 on day 1). For patients with non-squamous cell carcinoma, the preferred combination chemotherapy agent will be pemetrexed (500 mg/m^2^ on day 1), while for squamous cell carcinoma, the preferred combination agents will include docetaxel (135 mg/m^2^ on day 1) or albumin-bound paclitaxel (260 mg/m^2^ on day 1). Chemotherapy will be administered intravenously in three planned cycles, synchronized with radiation therapy during weeks 1, 4, and 7, with adjustments permitted based on patient tolerance.

### 2.5 Interventions

Participants in the experimental and control groups will receive JSG or placebo granules, respectively. Treatment will commence simultaneously with CCRT, administered orally three times daily (one dose per administration, to be taken with water), and will continue until the occurrence of SARIE or 2 weeks post-CCRT completion (week 9), whichever occurs first. In cases of Grade 1 ARIE, combined oral administration of mLDV will be initiated at a dose of 20 mL per administration, three times daily, and maintained until 2 weeks post-CCRT. No additional treatments will be provided before the onset of SARIE. If SARIE occurs, clinicians will provide supportive therapies as clinically indicated, which may include but are not limited to methylprednisolone, analgesics, intravenous infusion, or nasogastric tube feeding.

### 2.6 Investigational products

JSG is a compound TCM formulation composed of 12 Chinese medicinal materials and processed into granules using modern pharmaceutical procedures, including decoction, extraction, concentration, and drying. Its production follows the national standards of the Chinese Pharmacopeia and relevant standards for Chinese medicinal dispensing granules. Detailed information on these standards can be retrieved from the official website of the Chinese Pharmacopeia Commission^1^ using the corresponding standard numbers. The specific granule standards are as follows: *Gypsum Fibrosum* follows standard number SCYPBZ (PFKL)-2021277, with a crude drug-to-granule conversion ratio of 12.5:1; *Lonicerae Japonicae Flos* follows standard number YBZ-PFKL-2021073, with a crude drug-to-granule conversion ratio of 3:1; *Trichosanthis Radix* follows standard number YBZ-PFKL-2021121, with a crude drug-to-granule conversion ratio of 4.5:1; *Arnebiae Radix* follows standard number SCYPBZ (PFKL)-2023016, with a crude drug-to-granule conversion ratio of 10:1; *Glycyrrhizae Radix et Rhizoma* follows standard number YBZ-PFKL-2021049, with a crude drug-to-granule conversion ratio of 3:1; *Rehmanniae Radix* follows standard number YBZ-PFKL-2021115, with a crude drug-to-granule conversion ratio of 1.4:1; *Scrophulariae Radix* follows standard number YBZ-PFKL-2021131, with a crude drug-to-granule conversion ratio of 1.5:1; *Ophiopogonis Radix* follows standard number YBZ-PFKL-2022037, with a crude drug-to-granule conversion ratio of 1.1:1; *Glehniae Radix* follows standard number YBZ-PFKL-2022008, with a crude drug-to-granule conversion ratio of 2.5:1; *Bletillae Rhizoma* follows standard number JXYBZ-PFKL2024028, with a crude drug-to-granule conversion ratio of 5:1; *Typhae Pollen* follows standard number YBZ-PFKL-2023050, with a crude drug-to-granule conversion ratio of 5:1; and *Notoginseng Radix* follows standard number JSCYPBZ (PFKL)-2025014, with a crude drug-to-granule conversion ratio of 1.5:1.

Each sachet of JSG is composed of the above individual Chinese medicinal granules mixed according to the doses shown in Table 1. The compatibility ratio of the Chinese medicinal materials was determined based on previous clinical practice, and their crude drug equivalent doses are within the commonly used dosage ranges of the corresponding medicinal materials in the Chinese Pharmacopeia. Each sachet of JSG granules weighs 18.59 g, equivalent to 49 g of crude drugs. According to the finished product inspection reports of the individual Chinese medicinal dispensing granules, all individual granules used in JSG met the corresponding standards in quality control items, including description, thin-layer chromatography identification, and characteristic chromatogram, as well as relevant safety items, including microbial limit tests, heavy metals and harmful elements, and pesticide residues. Based on the legal quality standards for the medicinal materials used, the recommended dosage ranges in the Chinese Pharmacopeia, and the long-standing clinical use of JSG, the safety risks of JSG in the target population are considered manageable.

The placebo granules contain 5% JSG active components blended with an inert placebo base. The inclusion of 5% JSG active components in the placebo was mainly intended to improve sensory matching and help maintain blinding. A previous review on TCM placebo development reported that, to better simulate the special odor and taste of TCM preparations, some TCM placebos are designed to contain a low proportion of the parent drug, usually 5%–10% of the standard dose (27). The same review also noted that a previous study found no obvious pharmacological activity when less than 10% of the parent drug was added to the placebo formulation. It should be noted that residual pharmacological activity of the 5% JSG active components cannot be completely excluded. If present, such activity would be expected to attenuate the observed between-group treatment difference and lead to a more conservative estimate of efficacy. Therefore, the use of a placebo containing 5% JSG active components represents a pragmatic compromise between maintaining blinding feasibility and minimizing potential pharmacological interference from the placebo.

Both JSG and placebo granules will be indistinguishable in terms of mass, appearance, texture, odor, and taste. All granule preparations will be manufactured by Sichuan Neo-Green Pharmaceutical Technology Development Co., Ltd. in accordance with Good Manufacturing Practice requirements in China and the quality control standards of the Chinese Pharmacopeia.

The mLDV formulation consists of 250 mL of normal saline, 0.2 g of lidocaine, 10 mg of dexamethasone, and 2 mg of vitamin B12.

### 2.7 Primary efficacy outcomes

The primary outcomes are the incidence rates of ARIE and SARIE, defined as the proportion of subjects experiencing at least one episode of ≥Grade 1 ARIE and ≥Grade 3 ARIE, respectively, from the initiation of CCRT to 4 weeks post-CCRT completion (baseline to week 11). The grading of ARIE will be assessed using the Radiation Therapy Oncology Group (RTOG) scoring system (28), with evaluations conducted weekly.

### 2.8 Secondary efficacy outcomes

The secondary efficacy outcomes of the study include: (1) time to first occurrence of ARIE and SARIE; (2) severity of ARIE-related pain, assessed weekly using the Numerical Rating Scale (NRS); (3) severity of ARIE-related dysphagia, evaluated weekly using the NRS; (4) QoL, measured at baseline, week 3, week 5, week 7, week 9, and week 11 using the Functional Assessment of Cancer Therapy-Lung (FACT-L) questionnaire (29), which consists of a general cancer subscale (27 items) and a lung cancer-specific subscale (nine items), with each item scored on a 0–4 scale and a total score ranging from 0 to 144, where higher scores indicate better QoL; (5) short-term lung cancer efficacy, assessed at week 11 (4 weeks after CCRT completion) using computed tomography (CT) imaging to determine the objective response rate (ORR) and disease control rate (DCR) based on the Response Evaluation Criteria in Solid Tumors (RECIST) version 1.1 (30), with ORR calculated as the percentage of patients achieving either a complete response (CR) or partial response (PR) and DCR defined as the proportion of patients with CR, PR, or stable disease (SD); and (6) long-term lung cancer efficacy, including progression-free survival (PFS) and overall survival (OS). PFS is defined as the time from randomization to the first documented disease progression or death, whichever occurs first. OS is defined as the time from randomization to death from any cause. For PFS assessment, disease progression will be assessed using imaging examinations according to RECIST version 1.1 every 3 months during the first 3 years after the short-term efficacy assessment at week 11 and every 6 months during years 4–5, until disease progression, death, loss to follow-up, or completion of the 5-year follow-up period. For OS assessment, survival status will be collected every 3 months through outpatient visits, inpatient medical records, or telephone follow-up until death, loss to follow-up, or completion of the 5-year follow-up period.

### 2.9 Safety outcomes

Throughout the study, adverse events (AEs) and adverse reactions (ARs) will be meticulously monitored. AEs are defined as any undesirable medical occurrences that arise during the treatment period, which may present as symptoms, signs, diseases, or laboratory abnormalities. Laboratory assessments will include complete blood count, stool routine, urine routine, liver function tests, and renal function tests. These will be conducted weekly during the CCRT phase (0–7 weeks), and biweekly thereafter until the end of follow-up (week 11). All AEs will be promptly addressed with appropriate interventions, and comprehensive records will be maintained, documenting the time of occurrence, clinical manifestations, severity, treatment measures, outcomes, and the causal relationship with the investigational drug. The severity of AEs will be assessed using the Common Terminology Criteria for Adverse Events (CTCAE) version 5.0 (31), as established by the National Institutes of Health, with grades 3–5 categorized as serious adverse events (SAEs). In the event of an SAE, immediate emergency medical care will be provided, the study intervention will be discontinued, and a detailed report will be submitted to the ethics committee within 24 h. Participants will be closely monitored until the SAE is adequately managed. ARs are defined as AEs that are directly related to the investigational drug treatment. The causal relationship between the investigational drug and AEs will be assessed using the World Health Organization-Uppsala Monitoring Centre (WHO-UMC) system (32). AEs classified as “definitely related,” “probably related,” or “possibly related” will be considered ARs.

### 2.10 Mechanism outcomes

To systematically evaluate the potential mechanisms of action of JSG, the first 40 randomized participants at each center (160 in total) will provide blood and fecal samples at three time points: baseline, week 5 and week 9. These samples will be used to assess peripheral blood lymphocyte subsets, serum inflammatory cytokines, serum oxidative and antioxidative markers, gut microbiota, and serum/fecal metabolomic profiles. Fasting venous blood of 20 mL will be drawn from the antecubital vein between 6:00 a.m. and 9:00 a.m. Of this volume, 5 mL of whole blood will be used for lymphocyte subset analysis, and the remaining blood will be centrifuged to isolate serum. Both serum and fecal samples will be stored at −80 °C for subsequent batch analysis of mechanistic indicators.

Peripheral blood lymphocyte subsets will be determined via flow cytometry, including both relative and absolute counts of T lymphocytes (CD3+), helper T cells (CD3+CD4+), cytotoxic T cells (CD3+CD8+), B lymphocytes (CD19+), natural killer (NK) cells (CD16+CD56+), and the CD4+/CD8+ ratio. Serum inflammatory cytokines, including interferon-gamma (IFN-γ), interleukin-6 (IL-6), and tumor necrosis factor-alpha (TNF-α), will be quantified using enzyme-linked immunosorbent assays (ELISAs). Serum malondialdehyde (MDA) and superoxide dismutase (SOD) serve as biomarkers of oxidative stress and antioxidant capacity, respectively. MDA will be measured using an MDA assay kit based on the thiobarbituric acid method, while SOD will be detected using a T-SOD assay kit based on the hydroxylamine method (33).

Gut microbial profiles from fecal material will be characterized through 16S rRNA gene sequencing. Alpha diversity (within-sample diversity) will be examined using indices such as Shannon, Simpson, Chao1, and observed amplicon sequence variants, while beta diversity (between-sample differences) will be assessed using Bray–Curtis dissimilarity and visualized through principal coordinates analysis (34). To detect taxa with significant abundance differences among groups, the linear discriminant analysis effect size approach will be applied.

Serum metabolomic profiling will follow an untargeted liquid chromatography-mass spectrometry (LC-MS) strategy (35). Data will be interpreted using multivariate statistical methods, including principal component analysis and partial least squares discriminant analysis. Key metabolites contributing to group differentiation will be identified based on variable importance in projection scores. In parallel, short-chain fatty acids (SCFAs) in fecal samples will be quantified using an untargeted gas chromatography-mass spectrometry (GC-MS) approach (36).

### 2.11 Participant timeline

**FIGURE 2:**
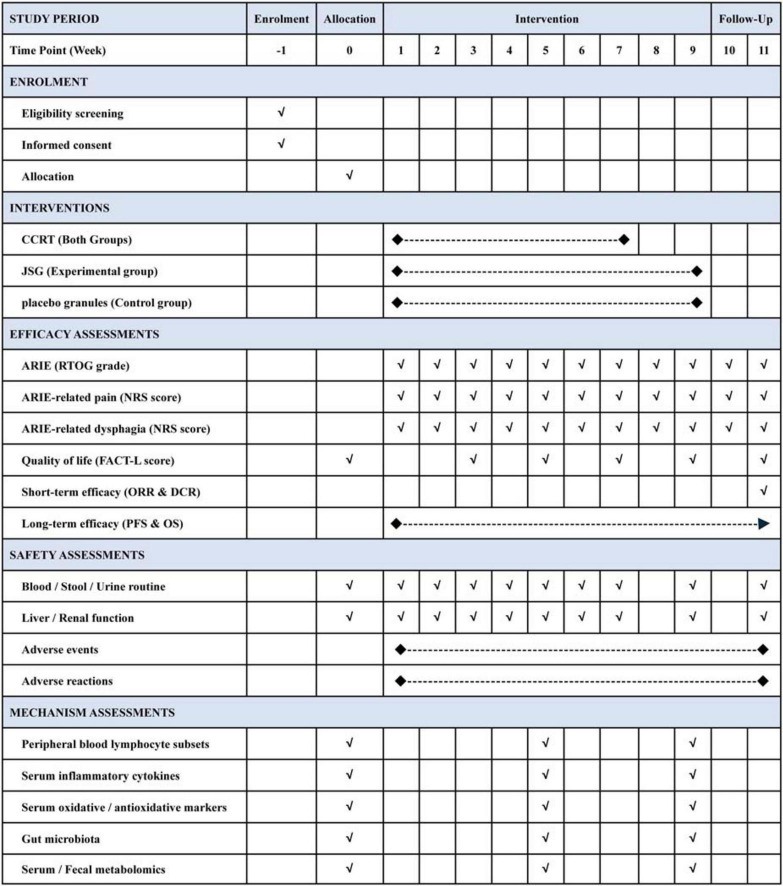
Detailed timeline of participant enrollment, interventions, and assessment schedule. ARIE, acute radiation-induced esophagitis; CCRT, concurrent chemoradiotherapy; DCR, disease control rate; FACT-L, Functional Assessment of Cancer Therapy-Lung; JSG, Jishi Shuanghua granules; NRS, numerical rating scale; ORR, objective response rate; OS, overall survival; PFS, progression-free survival; RTOG, Radiation Therapy Oncology Group.

The total study duration is 12 weeks, comprising a 1-week screening period, a 9-week treatment period, and a 2-week follow-up period. The detailed schedule of participant enrollment, interventions, and outcome assessments is illustrated in Figure 2.

### 2.12 Sample size estimation

The sample size estimation was based on the primary efficacy outcomes of this study (the incidence rates of ARIE and SARIE). According to a previous meta-analysis evaluating the effectiveness of a TCM formula using the “clearing heat” method, the estimated incidence rates in the control group were approximately 95% for ARIE and 25% for SARIE, with corresponding relative risks in the experimental group of 0.84 and 0.41, respectively (7). Sample size calculations were performed using PASS21 software, selecting the “Tests for Two Proportions” module. A two-sided α of 0.05, a power of 1−β = 0.80, and an expected dropout rate of 15% were assumed. The results indicated that the required sample size based on the ARIE incidence rate was 168 participants, while the sample size based on the SARIE incidence rate was 240 participants. To ensure sufficient statistical power for both primary outcomes, the final sample size was set at 240 participants, with 120 individuals allocated to each group.

### 2.13 Recruitment strategies

To successfully recruit sufficient participants and achieve the targeted sample size, a comprehensive recruitment strategy will be implemented across the four participating medical centers. Considering the scale, patient resources, and clinical conditions of each center, the planned recruitment targets include 80 participants at Sichuan Cancer Hospital, 60 at the Hospital of Chengdu University of TCM, 60 at West China Hospital of Sichuan University, and 40 at Leshan City Central District Cancer Hospital. The research team will identify potential participants through clinical referrals and targeted outreach, subsequently conducting face-to-face assessments and baseline evaluations to determine participant eligibility. Following ethical guidelines established by the Declaration of Helsinki, detailed explanations about the study’s purpose, intervention details, anticipated benefits, possible risks, procedures for handling and compensating study-related harm, privacy protection, and other essential information will be thoroughly provided. Participants will be enrolled only after they voluntarily provide fully informed written consent.

### 2.14 Randomization and blinding

Participants will be stratified by study center. An independent statistician will generate separate randomization sequences for each center using a computer-generated variable block randomization method (block sizes of 4, 6, and 8; allocation ratio 1:1). Allocation concealment will be achieved using sequentially numbered, opaque, sealed envelopes prepared in advance by an independent researcher. These envelopes will be securely stored by a third-party study monitor. After confirming participant eligibility and obtaining written informed consent, the designated research staff responsible for treatment allocation, who is not involved in any other part of the study, will open the envelopes in the order of enrollment to determine group assignment. Throughout the study, participants, healthcare providers, outcome assessors, and data analysts will remain blinded to treatment allocation. Unblinding will only be permitted after the completion of final data analysis or in emergency situations where immediate knowledge of the assigned intervention is required for clinical decision-making, and only with approval from the principal investigator.

### 2.15 Data collection and management

This study will collect comprehensive data, including the demographic characteristics of participants, baseline clinical information, treatment procedures, and all predefined outcome measures. Data will be recorded using standardized, paper-based case report forms (CRFs) specifically developed for this trial. Prior to study initiation, all research staff will receive centralized training on data collection procedures, CRF completion, assessment tool usage, and quality control measures to ensure consistency, accuracy, and reproducibility across study sites. To promote participant adherence and maximize follow-up completion rates, each participant will be provided with a clear visit schedule and follow-up instructions. Regular communication will be maintained throughout the study period, including reminder calls and psychological support when needed. For participants who drop out of the study, efforts will be made to collect key outcome data and to accurately document the timing and reasons for dropout, so that such data can be included in subsequent statistical analyses.

An electronic data capture (EDC) system will be utilized for data entry, management, and archiving. Data transcribed from paper CRFs will be entered into the system by trained personnel at each site. The EDC platform includes functionalities such as role-based access control, audit trail tracking, automatic logic and range checks, and real-time prompts for missing data, thereby ensuring completeness and integrity. To further ensure data quality, dual data entry and periodic review of submitted records will be implemented. Consistency validations and system-generated data queries will be issued regularly by the data management team and resolved promptly by site staff, with all corrections fully documented and auditable. Institutional ethics committees at each study center will conduct regular audits to monitor trial data, supervise protocol adherence, evaluate study progress, and ensure compliance with established standards. All original CRFs will be securely stored in locked cabinets under authorized supervision for a minimum of 5 years to maintain traceability and compliance with regulatory requirements. Electronic data will be encrypted and password-protected, accessible only to authorized study personnel, in full accordance with applicable data security and confidentiality standards.

### 2.16 Statistical analysis

All statistical analyses will be conducted based on the intention-to-treat (ITT) population, which includes all randomized participants. Descriptive statistics will be used to summarize baseline and outcome variables. Continuous variables with normal distribution will be presented as means and standard deviations, while those with non-normal distribution will be presented as medians and interquartile ranges. Categorical variables will be summarized as frequencies and percentages. The Shapiro–Wilk test will be used to assess the normality of continuous variables. For missing data, multiple imputation based on the Markov Chain Monte Carlo method will be performed under the assumption of missing at random.

For the primary efficacy outcomes (the incidence of ARIE and SARIE), between-group comparisons will be conducted using the Cochran–Mantel–Haenszel test, stratified by study center. Sensitivity analyses will include a pre-specified per-protocol (PP) analysis to assess the robustness of the findings. The PP population will consist of participants who complete the intervention and follow-up according to the protocol. Subgroup analyses will be performed to explore potential effect modifications by sex (male vs. female), age (≤55 vs. >55 years), body mass index (≤24 vs. >24), KPS score (70–80 vs. 90–100), histological subtype (non-squamous vs. squamous), and mean esophageal radiation dose (≤30 Gy vs. >30 Gy).

For other outcomes, repeated-measures continuous variables (such as NRS scores for pain and dysphagia, FACT-L quality of life scores, lymphocyte subpopulations, inflammatory cytokines, and oxidative and antioxidative biomarkers) will be analyzed using generalized estimating equation (GEE) models. These models will evaluate longitudinal changes between groups, with change from baseline as the dependent variable, and group, time, and group × time interaction as independent variables, adjusting for baseline values and study center. For the analyses of ARIE-related symptom outcomes, namely NRS scores for pain and dysphagia, the cumulative number of days of mLDV use up to each assessment will also be recorded and included as an additional time-varying covariate in the GEE models to account for the potential influence of rescue medication. Time-to-event variables (such as time to first occurrence of ARIE and SARIE, PFS, and OS) will be analyzed using Kaplan–Meier survival curves to estimate median event times. Cox proportional hazards models will be constructed to estimate hazard ratios, with study center included as a covariate. Categorical variables (such as ORR, DCR, and the incidence of AEs) will be analyzed using the chi-square test or Fisher’s exact test, as appropriate.

All statistical analyses will be performed using SPSS (version 28.0) and R (version 4.3.1). Two-sided significance testing will be applied. To control the type I error associated with the two primary efficacy outcomes, the Hochberg procedure will be used for multiplicity adjustment. Specifically, the *P*-values for the incidence rates of ARIE and SARIE will be ordered from smallest to largest. If the larger *P*-value is ≤0.05, both primary efficacy outcomes will be considered statistically significant. If the larger *P*-value is > 0.05 but the smaller *P*-value is ≤ 0.025, only the corresponding primary efficacy outcome will be considered statistically significant. If the smaller *P*-value is > 0.025, neither primary efficacy outcome will be considered statistically significant. No multiplicity adjustment will be applied to other outcomes or subgroup analyses, and the corresponding results will be considered exploratory.

### 2.17 Trial status

Recruitment for this clinical trial commenced in December 2025, with an anticipated completion date for participant enrollment in December 2028.

## 3 Discussion

This study was designed as a multicenter, randomized, double-blind, placebo-controlled trial, aiming to systematically evaluate the efficacy and potential mechanisms of JSG in reducing the risk and severity of ARIE in patients with NSCLC undergoing CCRT through rigorous clinical design and multi-omics analysis.

The pathogenesis of ARIE involves a complex biological process with multiple contributing factors, mainly including oxidative stress response, immune suppression, inflammatory cascade activation, and disruption of mucosal barrier integrity (37). Various potential radioprotective agents targeting these mechanisms have been proposed and subjected to clinical validation. Amifostine, a classical cytoprotective agent, exerts radioprotective effects by scavenging free radicals induced by ionizing radiation through its active metabolite WR-1065 (38). Early studies suggested that amifostine might reduce ARIE without compromising cancer control (39). However, a subsequent phase III clinical trial in patients with NSCLC receiving CCRT showed that although amifostine could alleviate dysphagia and pain to some extent, it failed to reduce the incidence of SARIE (40). Moreover, its use was associated with an increased risk of adverse events, such as nausea, vomiting, cardiac toxicity, and febrile neutropenia, which limited its clinical application (41).

Glutamine, another potential radioprotective agent, is believed to promote mucosal repair by serving as an energy substrate for gastrointestinal epithelial cells (42). A small pilot study initially demonstrated its potential in preventing ARIE (43). However, a subsequent double-blind, placebo-controlled trial in patients with NSCLC undergoing radiotherapy or CCRT was terminated due to interim analysis showing no significant improvement in the incidence and severity of ARIE (44). Additionally, non-steroidal anti-inflammatory drugs have also been investigated in the prevention of ARIE, but their efficacy remains unconfirmed (45). In recent years, the development of novel radioprotective agents has made certain progress. The prophylactic use of epigallocatechin-3-gallate and cystine-theanine compounds in phase II clinical trials has shown potential in reducing the severity of ARIE (6, 46), but further high-quality evidence is still required.

In this context, JSG, as a TCM formula, possesses multi-component, multi-target, and multi-pathway pharmacological characteristics, which are highly compatible with the complex and multi-step pathological process of ARIE. JSG is formulated based on the therapeutic principle of “clearing heat,” and its herbal components exert diverse pharmacological effects, including anti-inflammation, antioxidation, immune regulation, antibacterial, antiviral, mucosal repair, and hemostasis (Table 1), providing a novel therapeutic strategy for the prevention and treatment of ARIE.

Of particular interest, recent studies have indicated that gut microbiota dysbiosis and metabolic dysfunction play important roles in the development of ARIE. A study demonstrated that decreased abundance of beneficial bacteria capable of producing SCFAs, such as *Roseburia*, *Veillonella*, *Prevotella_9*, *Ruminococcus_2*, and *Megasphaera*, was associated with increased severity of ARIE (47). SCFAs, as essential metabolites of gut microbiota, possess important biological functions in regulating local immunity, exerting anti-inflammatory effects, and maintaining mucosal barrier integrity (48). Another study found that a TCM formula based on the “clearing heat” principle, Qinbai Qingfei Concentrate Pills, could modulate gut microbiota structure, promote SCFA production, and inhibit pulmonary inflammation, thus playing a therapeutic role in *Mycoplasma pneumonia* (49). Therefore, this study will further explore the regulatory effects of JSG on gut microbiota and metabolites through gut microbiota analysis and serum/fecal metabolomics, providing more comprehensive biological evidence for mechanistic research.

Moreover, it is noteworthy that whether JSG, as a potential radioprotective agent, would exert undesirable protective effects on tumor cells while protecting esophageal mucosal cells remains a critical scientific issue that deserves attention. Previous studies have demonstrated that multiple components of JSG, such as *Lonicerae Japonicae Flos* (9)*, Arnebiae Radix* (11)*, Glycyrrhizae Radix et Rhizoma*(13)*, Rehmanniae Radix* (14)*, Ophiopogonis Radix* (17)*, Glehniae Radix* (18)*, and Notoginseng Radix* (23), possess anticancer properties, which may exert mucosal protective effects without negatively impacting, and possibly even synergistically enhancing, the antitumor efficacy of CCRT. Therefore, this study will also comprehensively evaluate the impact of JSG on both short-term efficacy (ORR, DCR) and long-term efficacy (PFS, OS) of CCRT in patients with NSCLC, to fully assess its clinical value and safety.

Although this study was designed as a multicenter, randomized, double-blind, placebo-controlled trial with robust methodological strengths, several limitations should be acknowledged. First, patients enrolled in this study were primarily from Sichuan Province, China. Given regional characteristics, lifestyle habits, and genetic backgrounds, the representativeness and generalizability of the results are limited, and further validation in other regions and larger populations is warranted. Second, although this study preliminarily explored the potential mechanisms of JSG through multi-omics strategies, it remains difficult to comprehensively elucidate the key active components and core action pathways of JSG in the prevention and treatment of ARIE. Further in-depth elucidation of specific signaling pathways, target proteins, and cellular effects through basic and translational research is needed, which will be an important direction for future studies.

In conclusion, this study responds to the current lack of rigorous clinical evidence regarding the use of JSG for ARIE by generating comprehensive data from both clinical and mechanistic perspectives. Through a combination of randomized controlled trial design and multiomics analyses, it aims to construct an integrated evidence chain that elucidates the efficacy, safety, and biological basis of JSG. The findings are expected to provide a scientific rationale for the modern translational application of the “clearing heat” principle in ARIE management and to support its broader implementation in evidence-based oncology care.

## Footnotes

1
https://www.chp.org.cn/
