# Supplementary material for: Efficacy of Jishi Shuanghua Granules in preventing radiation-induced esophagitis in non-small cell lung cancer patients undergoing concurrent chemoradiotherapy: protocol for a multicenter, randomized, double-blind, placebo-controlled trial
Source: Front Med (Lausanne). 2026 Jun 23;13:1814342. doi: 10.3389/fmed.2026.1814342 (PMC13337399; doi:10.3389/fmed.2026.1814342)
Supplement: Supplementary file 1 [file Supplementary_file_1.pdf]

SPIRIT Checklist: Recommended Items to Address in a Clinical Trial Protocol and Related Documents

**Section/Item**<br>**Administrative information**	**Item**<br>**Number**	**Description**
Title	1	Descriptive title identifying the study design, population, interventions, and, if applicable, trial acronym<br>Efficacy of Jishi Shuanghua Granules in Preventing Radiation-Induced Esophagitis in Non-Small Cell Lung Cancer Patients Undergoing Concurrent Chemoradiotherapy: Protocol for a Multicenter, Randomized, Double-Blind, Placebo-Controlled Trial
Trial registration	2a	Trial identifier and registry name. If not yet registered, name of intended registry.<br>http://itmctr.ccebtcm.org.cn/, identifier ITMCTR2025000641
	2b	All items from the World Health Organization Trial Registration Data Set (Appendix Table, available at www.annals.org)<br>Not applicable for this trial.
Protocol version	3	Date and version identifier<br>Date August 3, 2025 and version 2.0
Funding	4	Sources and types of financial, material, and other support<br>This work was supported by the Chengdu Science and Technology Bureau (Grant No. 2024-YF05-02230-SN and 2024-YF05-01283-SN), the Scientific Research Project of the Sichuan Medical Association (Grant No. 2024HR15),

		the Sichuan Provincial Administration of Traditional Chinese Medicine Research Special Fund (Grant No. 2024MS4205), the Sichuan Cancer Hospital Key Project Fund for Radiotherapy in Integrated Chinese and Western Medicine (Grant No. YB2025062003), the Chengdu Medical Research Project (Grant No. 2025144), and the Chengdu University of Traditional Chinese Medicine and Chengdu Health Commission Joint Innovation Fund (Grant No. WXLH202501260). The funders had no role in the study design, data collection and analysis, interpretation of results, or manuscript preparation.
Roles and responsibilities	5a	Names, affiliations, and roles of protocol contributors<br><br>Huakang Li [1,2,#], Yuanzhen Mi [1,2,#], Ke Xu [2,#], Ming Fan [2,#], Jun Yin [2,3,#], Qiang Li [4,#], Ziliang Wu [1], Cuicui Gong [1], Yunjing Jia[1], Pengxuan Gu [1], Shanshan Wei [2], Zhonglin Zhang [2], Shuo Zhang [2], Yuanyuan Zheng [5], Bing Lin [6,*], Jinyi Lang [1,2*], Biao Zhao [7,*], Meihua Chen [2,*]<br><br>[1] School of Clinical Medicine, Chengdu University of Traditional Chinese Medicine, Chengdu, Sichuan, China<br>[2] Department of Radiation Oncology, Radiation Oncology Key Laboratory of Sichuan Province, Sichuan Clinical Research Center for Cancer, Sichuan Cancer Hospital & Institute, Sichuan Cancer Center, Affiliated Cancer Hospital of University of Electronic Science and Technology of China, Chengdu, China

		[3] Department of Medical Oncology, Affiliated Hospital of Sichuan Nursing Vocational College, The Third People's Hospital of Sichuan Province, Chengdu, Sichuan, China.<br>[4] Department of Oncology, Hospital of Chengdu University of Traditional Chinese Medicine, Chengdu, China.<br>[5] Department of Integrated Traditional Chinese and Western Medicine, Internal Medicine, Leshan City Shizhong District Cancer Hospital, Leshan, Sichuan, China.<br>[6] Health Management Center, Hospital of Chengdu University of Traditional Chinese Medicine, Chengdu, China.<br>[7] Department of Integrative Chinese and Western Medicine, Sichuan Cancer Hospital & Institute, Sichuan Cancer Center, Affiliated Cancer Hospital of the University of Electronic Science and Technology of China, Chengdu, China.<br>.
	5b	Name and contact information for the trial sponsor<br>Chengdu Technology Bureau, No. 2, Caoshi Street, Qingyang District, Chengdu 610017, Sichuan Province, China. Phone: +86 28-86924834.<br>Sichuan Medical Association, No. 818, Xing Shi Road, Wuhou District, Chengdu 610043, Sichuan Province, China. Phone: +86 28-861371412.<br>Sichuan Provincial Administration of Traditional Chinese Medicine, No. 155, Taisheng South Road, Qingyang District, Chengdu 610020, Sichuan Province, China. Phone: +86 28-86780331.

	5c	Role of study sponsor and funders, if any, in study design; collection, management, analysis, and interpretation of data; writing of the report; and the decision to submit the report for publication, including whether they will have ultimate authority over any of these activities<br>Funders of this study have no role in any abovementioned activities.
	5d	Composition, roles, and responsibilities of the coordinating center, steering committee, end point adjudication committee, data management team, and other individuals or groups overseeing the trial, if applicable (see item 21a for DMC)<br>The Research Ethics Committee of Sichuan Cancer Hospital, Hospital of Chengdu University of Traditional Chinese Medicine, West China Hospital of Sichuan University, and Leshan City Shizhong District Cancer Hospital will perform regular audits to verify data accuracy.
**Introduction**		
Background and rationale	6a	Description of research question and justification for undertaking the trial, including summary of relevant studies (published and unpublished) examining benefits and harms for each intervention<br>Acute radiation-induced esophagitis (ARIE) is a common complication in non-small cell lung cancer (NSCLC) patients undergoing concurrent chemoradiotherapy (CCRT), significantly impacting their quality of life and treatment outcomes. Although various radioprotective agents have been explored, there is still no definitive, effective treatment for preventing or managing ARIE. Most current approaches focus on symptom management, with limited efficacy

		and potential side effects.<br><br>Jishi Shuanghua Granules (JSG), a Traditional Chinese Medicine formulation based on the "clearing heat" theory, has been widely used in clinical practice for ARIE, showing positive feedback. However, high-quality clinical evidence supporting its effectiveness remains scarce. This trial aims to evaluate the efficacy of JSG in reducing the risk and severity of ARIE in NSCLC patients undergoing CCRT and to explore its underlying mechanisms through multi-omics analysis.<br>This study will provide high-quality evidence to support JSG's use in ARIE prevention and treatment, filling a critical gap in the current ARIE management strategies.
	6b	Explanation for choice of comparators<br>In this trial, the comparator group will receive a placebo, which serves as a standard control to assess the efficacy of Jishi Shuanghua Granules (JSG) in reducing the risk and severity of acute radiation-induced esophagitis (ARIE). The placebo is selected to eliminate any potential bias in evaluating the true effects of JSG, ensuring that any observed benefits are specifically due to the intervention itself, rather than the psychological or physiological effects of receiving treatment.
Objectives	7	Specific objectives or hypotheses<br>The primary objective is to evaluate the efficacy of Jishi Shuanghua Granules (JSG) in reducing the incidence and

		severity of acute radiation-induced esophagitis (ARIE) in NSCLC patients undergoing CCRT, as measured by the incidence of ARIE (≥ Grade 1) and severe ARIE (≥ Grade 3).<br><br>Secondary objectives include assessing time to first occurrence of ARIE, ARIE-related pain and dysphagia, quality of life, and short-term lung cancer efficacy (objective response rate and disease control rate).<br>Mechanistic objectives aim to explore the effects of JSG on biomarkers such as lymphocyte subsets, inflammatory cytokines, oxidative stress markers, gut microbiota, and metabolomic profiles.<br>The hypotheses are that JSG will significantly reduce ARIE severity, improve quality of life, and produce measurable biological changes in biomarkers, indicating its underlying mechanisms.
Trial design	8	Description of trial design, including type of trial (e.g., parallel group, crossover, factorial, single group), allocation ratio, and framework (e.g., superiority, equivalence, noninferiority, exploratory)<br>The study is designed as a multicenter, prospective, randomized, double-blind, placebo-controlled clinical trial with two parallel groups.
**Methods**		
Participants, interventions, and outcomes		
Study setting	9	Description of study settings (e.g., community clinic, academic hospital) and list of countries where data will be collected. Reference to where list of study sites can be obtained

		This study will be conducted in four academic hospitals in China, including Sichuan Cancer Hospital, Hospital of Chengdu University of Traditional Chinese Medicine, West China Hospital of Sichuan University, and Leshan City Central District Cancer Hospital. The trial will be conducted exclusively in China. A full list of study sites and their contact information is available upon request from the corresponding author.
Eligibility criteria	10	Inclusion and exclusion criteria for participants. If applicable, eligibility criteria for study centers and individuals who will perform the interventions (e.g., surgeons, psychotherapists)<br>**Inclusion criteria**<br>(1) age between 18 and 75 years; (2) pathologically confirmed NSCLC, classified as stage III (according to the 8th edition of the American Joint Committee on Cancer staging system); (3) clinically assessed as unresectable and scheduled to undergo definitive CCRT; (4) mean esophageal radiation dose > 20 Gy; (5) normal hematologic, hepatic, and renal function (based on laboratory reference ranges); and (6) Karnofsky Performance Status (KPS) score ≥ 70.<br><br>**Exclusion criteria**<br>(1) prior history of thoracic radiotherapy; (2) pregnancy or lactation; (3) inability to maintain adequate oral intake; and (4) history of esophageal diseases, including but not limited to esophageal cancer, active reflux esophagitis, or esophageal reconstruction.
Interventions	11a	Interventions for each group with sufficient detail to allow replication, including how and when they will be

		administered<br>Participants in the experimental and control groups will receive JSG or placebo granules, respectively. Treatment will commence simultaneously with CCRT, administered orally three times daily (one dose per administration, to be taken with water), and will continue until the occurrence of SARIE or two weeks post-CCRT completion (week 9), whichever occurs first. In cases of Grade 1 ARIE, combined oral administration of mLDV will be initiated at a dose of 20 mL per administration, three times daily, and maintained until two weeks post-CCRT. No additional treatments will be provided before the onset of SARIE. If SARIE occurs, clinicians will provide supportive therapies as clinically indicated, which may include but are not limited to methylprednisolone, analgesics, intravenous infusion, or nasogastric tube feeding.
	11b	Criteria for discontinuing or modifying allocated interventions for a given trial participant (e.g., drug dose change in response to harms, participant request, or improving/worsening disease)<br>(1) Participants have the right to voluntarily withdraw from the trial at any stage for any reason;<br>(2) Participants who experience severe adverse events that preclude continuation in the trial;<br>(3) Death.
	11c	Strategies to improve adherence to intervention protocols, and any procedures for monitoring adherence (e.g., drug tablet return, laboratory tests)<br>To enhance adherence to intervention protocols and ensure effective monitoring, we will implement several key

		strategies: delivering clear communication and education to help participants understand the importance of protocol compliance, conducting regular follow-ups for ongoing support, and offering small incentives to encourage consistent participation. Together, these strategies are designed to improve adherence, minimize deviations, and ensure the reliability of study outcomes.
	11d	Relevant concomitant care and interventions that are permitted or prohibited during the trial<br>Standard CCRT is permitted for all participants. mLDV is allowed after Grade 1 ARIE onset in both groups. Use of other Traditional Chinese Medicine, herbal remedies, or radioprotective agents before ARIE onset is prohibited. Supportive care (e.g., analgesics, steroids, nutrition) is permitted after severe ARIE occurs.
Outcomes	12	Primary, secondary, and other outcomes, including the specific measurement variable (e.g., systolic blood pressure), analysis metric (e.g., change from baseline, final value, time to event), method of aggregation (e.g., median, proportion), and time point for each outcome. Explanation of the clinical relevance of chosen efficacy and harm outcomes is strongly recommended<br>The primary outcomes of this study are the incidence of acute radiation-induced esophagitis (ARIE, ≥ Grade 1) and severe ARIE (SARIE, ≥ Grade 3), assessed using the Radiation Therapy Oncology Group (RTOG) criteria. These will be measured as the proportion of patients who develop ARIE or SARIE from the start of concurrent chemoradiotherapy (CCRT) to four weeks after its completion (week 11). These outcomes are clinically important as they directly reflect the effectiveness of Jishi Shuanghua Granules (JSG) in preventing treatment-related esophageal

		toxicity, which can interrupt cancer therapy and significantly impair patient quality of life.<br><br>Secondary outcomes include time to first occurrence of ARIE and SARIE, evaluated as time-to-event variables; ARIE-related pain and dysphagia, measured weekly using the Numerical Rating Scale (NRS) and analyzed as changes from baseline; and quality of life, assessed using the Functional Assessment of Cancer Therapy–Lung (FACT-L) questionnaire at multiple time points, with total scores aggregated as means. Additional secondary outcomes involve tumor response, including objective response rate (ORR) and disease control rate (DCR) at week 11, based on RECIST 1.0 criteria. Long-term outcomes such as progression-free survival (PFS) and overall survival (OS) will be assessed over the follow-up period and analyzed using survival analysis methods.<br><br>Other exploratory outcomes include mechanistic indicators related to immune function, inflammation, oxidative stress, gut microbiota, and metabolism. These will be evaluated through multi-omics analyses of blood and fecal samples collected at baseline, week 5, and week 9. Key biomarkers include lymphocyte subsets (via flow cytometry), inflammatory cytokines (e.g., IL-6, TNF-α, IFN-γ), oxidative stress markers (e.g., MDA, SOD), microbial diversity and composition (via 16S rRNA sequencing), and metabolomic profiles (via LC-MS and GC-MS). These outcomes aim to explore the biological mechanisms underlying JSG's potential protective effects and support its integration into evidence-based cancer care.

Participant timeline	13	Time schedule of enrollment, interventions (including any runins and washouts), assessments, and visits for participants. A schematic diagram is highly recommended (Figure).<br>As shown in figure 1 and figure 2.
Sample size	14	Estimated number of participants needed to achieve study objectives and how it was determined, including clinical and statistical assumptions supporting any sample size calculations<br>The sample size estimation was based on the primary efficacy outcomes of this study (the incidence rates of ARIE and SARIE). According to a previous meta-analysis evaluating the effectiveness of a TCM formula using the “clearing heat” method, the estimated incidence rates in the control group were approximately 95% for ARIE and 25% for SARIE, with corresponding relative risks in the experimental group of 0.84 and 0.41, respectively. Sample size calculations were performed using PASS21 software, selecting the "Tests for Two Proportions" module. A two-sided α of 0.05, a power of $1-\beta = 0.80$, and an expected dropout rate of 15% were assumed. The results indicated that the required sample size based on the ARIE incidence rate was 168 participants, while the sample size based on the SARIE incidence rate was 240 participants. To ensure sufficient statistical power for both primary outcomes, the final sample size was set at 240 participants, with 120 individuals allocated to each group.
Recruitment	15	Strategies for achieving adequate participant enrollment to reach target sample size<br>To successfully recruit sufficient participants and achieve the targeted sample size, a comprehensive recruitment strategy will be implemented across the four participating medical centers. Considering the scale, patient resources,

		and clinical conditions of each center, the planned recruitment targets include 80 participants at Sichuan Cancer Hospital, 60 at the Hospital of Chengdu University of Traditional Chinese Medicine, 60 at West China Hospital of Sichuan University, and 40 at Leshan City Central District Cancer Hospital. The research team will identify potential participants through clinical referrals and targeted outreach, subsequently conducting face-to-face assessments and baseline evaluations to determine participant eligibility. Following ethical guidelines established by the Declaration of Helsinki, detailed explanations about the study's purpose, intervention details, anticipated benefits, possible risks, procedures for handling and compensating study-related harm, privacy protection, and other essential information will be thoroughly provided. Participants will be enrolled only after they voluntarily provide fully informed written consent.
Assignment of interventions (for controlled trials)		
Allocation Sequence generation	16a	Method of generating the allocation sequence (e.g., computer-generated random numbers), and list of any factors for stratification. To reduce predictability of a random sequence, details of any planned restriction (e.g., blocking) should be provided in a separate document that is unavailable to those who enroll participants or assign interventions.<br>Participants will be stratified by study center. An independent statistician will generate separate randomization sequences for each center using a computer-generated variable block randomization method (block sizes of 4, 6, and 8; allocation ratio 1:1).
Allocation concealment	16b	Mechanism of implementing the allocation sequence (e.g., central telephone; sequentially numbered, opaque, sealed

mechanism		envelopes), describing any steps to conceal the sequence until interventions are assigned<br>Allocation will be implemented using sequentially numbered, opaque, sealed envelopes stored by a third-party monitor. After consent and eligibility confirmation, designated staff will open the next envelope in order, ensuring allocation concealment until assignment.
Implementation	16c	Who will generate the allocation sequence, who will enroll participants, and who will assign participants to interventions<br>The allocation sequence will be generated by an independent statistician using computer-based randomization. Participants will be enrolled by trained research staff at each study site. Group assignments will be conducted by designated personnel not involved in other study procedures, using sealed envelopes to ensure blinding and allocation concealment.
Blinding (masking)	17a	Who will be blinded after assignment to interventions (e.g., trial participants, care providers, outcome assessors, data analysts), and how<br>After assignment, trial participants, care providers, outcome assessors, and data analysts will all be blinded to group allocation. Jishi Shuanghua Granules (JSG) and placebo granules are identical in appearance, taste, and packaging. Group codes will be kept confidential by a third-party monitor and will only be unblinded after final data analysis or in case of medical emergencies, with approval from the principal investigator.
	17b	If blinded, circumstances under which unblinding is permissible, and procedure for revealing a participant's allocated

		intervention during the trial<br>Unblinding is only permissible in emergency situations where knowledge of the assigned intervention is essential for clinical management. In such cases, the treating physician must submit a formal request to the principal investigator. Upon approval, the third-party study monitor will disclose the participant's group allocation. All unblinding events will be documented, including the reason, time, and personnel involved. Routine unblinding will not occur until after final data analysis is complete.
Data collection, management, and analysis		
Data collection methods	18a	Plans for assessment and collection of outcome, baseline, and other trial data, including any related processes to promote data quality (e.g., duplicate measurements, training of assessors) and a description of study instruments (e.g., questionnaires, laboratory tests) along with their reliability and validity, if known. Reference to where data collection forms can be found, if not in the protocol.<br>Outcome, baseline, and other trial data will be collected using standardized, paper-based case report forms (CRFs) designed specifically for this study. Data collection will include demographic information, clinical history, treatment details, primary and secondary outcomes, and safety indicators. All outcome assessments will be conducted by trained personnel following standardized procedures to ensure consistency across sites.<br>Key instruments include the Radiation Therapy Oncology Group (RTOG) criteria for grading ARIE, the Numerical Rating Scale (NRS) for pain and dysphagia, and the Functional Assessment of Cancer Therapy–Lung (FACT-L)

		questionnaire for quality of life. These tools are widely validated and commonly used in oncology trials. Laboratory outcomes such as blood tests and cytokine levels will be measured using validated assays, including ELISA and flow cytometry.<br><br>To promote data quality, all assessors will undergo centralized training before the trial starts. Duplicate data entry and regular monitoring will be implemented to ensure accuracy and completeness. An electronic data capture (EDC) system will be used for data management, including real-time error checking and audit trails. Data collection forms are available upon request from the study sponsor or corresponding author.
	18b	Plans to promote participant retention and complete follow-up, including list of any outcome data to be collected for participants who discontinue or deviate from intervention protocols<br>Regular communication will be maintained throughout the study period, including reminder calls and psychological support when needed. For participants who drop out of the study, efforts will be made to collect key outcome data and to accurately document the timing and reasons for dropout, so that such data can be included in subsequent statistical analyses.
Data management	19	Plans for data entry, coding, security, and storage, including any related processes to promote data quality (e.g., double data entry; range checks for data values). Reference to where details of data management procedures can be found, if not in the protocol.<br><br>Data will be entered from paper CRFs into a secure electronic system using double data entry. Range and logic checks will ensure data accuracy. Each participant will have a unique ID to protect confidentiality. Data will be stored on password-protected servers with restricted access and audit trails. Full data management procedures are available upon request.

Statistical methods	20a	Statistical methods for analyzing primary and secondary outcomes. Reference to where other details of the statistical analysis plan can be found, if not in the protocol.<br><br>Primary outcomes (incidence of ARIE and SARIE) will be analyzed using the Cochran–Mantel–Haenszel test, stratified by study center. Secondary outcomes such as symptom scores and quality of life will be analyzed using generalized estimating equations (GEE) to assess group differences over time. Time-to-event outcomes (e.g., PFS, OS) will be analyzed using Kaplan–Meier curves and Cox proportional hazards models. A hierarchical testing strategy will be used for the two primary outcomes to control type I error.
	20b	Methods for any additional analyses (e.g., subgroup and adjusted analyses)<br>Predefined subgroup analyses will be conducted based on variables such as sex, age, BMI, KPS score, tumor histology, and esophageal radiation dose. Adjusted analyses may be performed using multivariable models to control for baseline imbalances where appropriate. These analyses are exploratory and intended to assess consistency of the treatment effect across subgroups.
	20c	Definition of analysis population relating to protocol nonadherence (e.g., as-randomized analysis), and any statistical methods to handle missing data (e.g., multiple imputation)<br>The primary analysis will follow the intention-to-treat (ITT) principle, including all randomized participants. A per-protocol (PP) analysis will be conducted as a sensitivity analysis. Missing data will be addressed using multiple imputation under the assumption of missing at random, with the Markov Chain Monte Carlo method applied where applicable.

Monitoring		
Data monitoring	21a	Composition of DMC; summary of its role and reporting structure; statement of whether it is independent from the sponsor and competing interests; and reference to where further details about its charter can be found, if not in the protocol. Alternatively, an explanation of why a DMC is not needed.<br>The Research Ethics Committees of Sichuan Cancer Hospital, Hospital of Chengdu University of Traditional Chinese Medicine, West China Hospital of Sichuan University, and Leshan City Shizhong District Cancer Hospital will perform regular independent audits. These include verifying data accuracy, monitoring trial conduct, reviewing study progress, and ensuring compliance with ethical standards and protocol requirements. These committees operate independently from the sponsor and study team, with no competing interests.
	21b	Description of any interim analyses and stopping guidelines, including who will have access to these interim results and make the final decision to terminate the trial.<br>No interim analyses are planned for this trial. The study will proceed as scheduled unless significant safety concerns arise. In such cases, the principal investigator and ethics committees will review the situation and decide whether to modify or terminate the trial.
Harms	22	Plans for collecting, assessing, reporting, and managing solicited and spontaneously reported adverse events and other unintended effects of trial interventions or trial conduct<br>Throughout the study, adverse events (AEs) and adverse reactions (ARs) will be meticulously monitored. AEs are defined as any undesirable medical occurrences that arise during the treatment period, which may present as

		symptoms, signs, diseases, or laboratory abnormalities. Laboratory assessments will include complete blood count, stool routine, urine routine, liver function tests, and renal function tests. These will be conducted weekly during the CCRT phase (0-7 weeks), and biweekly thereafter until the end of follow-up (week 11). All AEs will be promptly addressed with appropriate interventions, and comprehensive records will be maintained, documenting the time of occurrence, clinical manifestations, severity, treatment measures, outcomes, and the causal relationship with the investigational drug. The severity of AEs will be assessed using the Common Terminology Criteria for Adverse Events (CTCAE) version 5.0, as established by the National Institutes of Health, with grades 3-5 categorized as serious adverse events (SAEs). In the event of an SAE, immediate emergency medical care will be provided, the study intervention will be discontinued, and a detailed report will be submitted to the ethics committee within 24 hours. Participants will be closely monitored until the SAE is adequately managed. ARs are defined as AEs that are directly related to the investigational drug treatment. The causal relationship between the investigational drug and AEs will be assessed using the World Health Organization-Uppsala Monitoring Centre (WHO-UMC) system. AEs classified as "definitely related," "probably related," or "possibly related" will be considered ARs.
Auditing	23	Frequency and procedures for auditing trial conduct, if any, and whether the process will be independent from investigators and the sponsor<br>Regular audits of trial conduct will be carried out by the Research Ethics Committees of the participating centers. These audits will assess protocol compliance, data accuracy, participant safety, and overall study progress. Audits will be conducted approximately every six months . The auditing process is entirely independent from the investigators and the study sponsor to ensure objectivity and integrity.
**Ethics and dissemination**		

Research ethics approval	24	Plans for seeking REC/IRB approval<br>The study protocol was approved by the Research Ethics Committee of Leshan City Shizhong District Cancer Hospital on March 4, 2025 (approval number: EC-2025-001). The ethical approval is recognized by the other three participating centers.
Protocol amendments	25	Plans for communicating important protocol modifications (e.g., changes to eligibility criteria, outcomes, analyses) to relevant parties (e.g., investigators, RECs/IRBs, trial participants, trial registries, journals, regulators)<br>Any important protocol modifications (e.g., changes to eligibility criteria, outcomes, or analysis methods) will be submitted for review and approval by the Research Ethics Committees of all participating centers, as well as the International Traditional Medicine Clinical Trial Registry. Approved changes will be promptly communicated to investigators, trial participants (if relevant), and updated in trial registries and future publications, in accordance with regulatory and ethical guidelines.
Consent or assent	26a	Who will obtain informed consent or assent from potential trial participants or authorized surrogates, and how (see item 32)<br>Informed consent will be obtained by trained research staff at each study site prior to any trial-related procedures. Staff will explain the study purpose, procedures, risks, benefits, and participant rights using a standardized consent form. Consent discussions will be conducted face-to-face in a private setting, allowing ample time for questions. Written informed consent will be required from all participants or their legally authorized representatives before

		enrollment.
	26b	Additional consent provisions for collection and use of participant data and biological specimens in ancillary studies, if applicable<br>Not applicable for this trial.
Confidentiality	27	How personal information about potential and enrolled participants will be collected, shared, and maintained in order to protect confidentiality before, during, and after the trial<br>All original CRFs will be securely stored in locked cabinets under authorized supervision for a minimum of five years to maintain traceability and compliance with regulatory requirements. Electronic data will be encrypted and password-protected, accessible only to authorized study personnel, in full accordance with applicable data security and confidentiality standards.
Declaration of interests	28	Financial and other competing interests for principal investigators for the overall trial and each study site<br>None.
Access to data	29	Statement of who will have access to the final trial data set, and disclosure of contractual agreements that limit such access for investigators<br>Access to the final trial dataset will be limited to the principal investigator, and designated members of the research team. There are no contractual agreements that restrict investigators' access to the full dataset. All data analyses and publications will be conducted independently by the research team without sponsor interference.

Ancillary and post-trial care	30	Provisions, if any, for ancillary and post-trial care, and for compensation to those who suffer harm from trial participation<br>Participants who experience harm related to trial participation will receive appropriate medical care at no cost. Each study center is responsible for managing and reporting adverse events, and compensation will be provided in accordance with local regulations and institutional policies.
Dissemination policy	31a	Plans for investigators and sponsor to communicate trial results to participants, health care professionals, the public, and other relevant groups (e.g., via publication, reporting in results databases, or other data-sharing arrangements), including any publication restrictions<br>Upon reasonable request, the data sets generated and/or analyzed can be obtained from the corresponding authors. The results of this study will be published in open-access and peer-reviewed journals.
	31b	Authorship eligibility guidelines and any intended use of professional writers<br>We haven't used such a service.
	31c	Plans, if any, for granting public access to the full protocol, participant-level data set, and statistical code<br>This was already mentioned in 18a and 31a.
**Appendices**		
Informed consent materials	32	Model consent form and other related documentation given to participants and authorized surrogates<br>The model consent form and other relevant documentation provided to participants and authorized surrogates are

		available from the corresponding author upon reasonable request.
Biological specimens	33	Plans for collection, laboratory evaluation, and storage of biological specimens for genetic or molecular analysis in the current trial and for future use in ancillary studies, if applicable<br>Blood and fecal samples from participants will be collected and stored at -80°C for final analysis.
